# Microspectrometer-Enabled Real-Time Concentration Monitoring in the Microfluidic Protein Enrichment Chip

**DOI:** 10.3390/bios15010001

**Published:** 2024-12-24

**Authors:** Dong-Li Li, Wen-Shu Huang, Yi Hung Wu, Chun-Ping Jen

**Affiliations:** 1 Fisheries College, Jimei University, Xiamen 361021, China; donglili@jmu.edu.cn (D.-L.L.); wshuang@jmu.edu.cn (W.-S.H.); 2 Engineering Research Center of the Modern Technology for Eel Industry, Ministry of Education, Xiamen 361021, China; 3 Department of Mechanical Engineering and Advanced Institute of Manufacturing for High-Tech Innovations, National Chung Cheng University, Chia-Yi 62102, Taiwan; wuyihung02@gmail.com; 4 School of Dentistry, College of Dental Medicine, Kaohsiung Medical University, Kaohsiung 80708, Taiwan

**Keywords:** microfluidic chips, protein detection, microspectrometer, fluorescence, preconcentration

## Abstract

This study presents a novel microspectrometer-integrated microfluidic system for real-time protein concentration monitoring. The device employs electrokinetic principles for efficient protein preconcentration in a PDMS and Nafion film channel. Using FITC-labeled BSA as a model protein, the system demonstrated a linear correlation between protein concentration and absorbance at 491 nm. Notably, it achieved a 833-fold concentration increase from an initial 10 nM within 20 min. The compact microspectrometer system offers enhanced accuracy and sensitivity compared to traditional fluorescence microscopy methods. This innovation presents a promising solution for portable and point-of-care diagnostic applications, facilitating timely disease detection and monitoring. The findings highlight the potential for this technology to advance protein analysis and biomarker discovery in clinical settings, potentially improving patient outcomes through enhanced diagnostic capabilities.

## 1. Introduction

Protein detection plays a crucial role in early disease diagnosis, offering valuable insights into health status and enabling timely interventions [1,2,3]. Proteomic technologies have revolutionized the identification of protein biomarkers for various diseases, facilitating early detection, treatment monitoring, and therapy target identification [4]. Advanced analytical techniques, such as digital immunoassays and optical-based micro- and nano-biosensors, have significantly enhanced the detection of low-abundance proteins. This progress has paved the way for ultrasensitive disease diagnostics and the identification of rare proteins. These advancements have led to the detection of disease-specific proteins, such as Galectin-3, which serve as biomarkers for conditions including cancer, cardiovascular diseases, and diabetes. This underscores the significance of protein detection in early disease diagnosis and its potential to save lives. Microfluidic technology plays a pivotal role in protein manipulation, particularly in concentration and enrichment processes, as proteins cannot be amplified like DNA using PCR techniques. This technology offers innovative solutions for protein analysis and detection, which are key molecules in numerous biological processes [5]. By incorporating protein preconcentration platforms into microfluidic channels, researchers can achieve stable depletion and enrichment modes, facilitating the trapping and concentration of proteins at designated zones within the channels. The evolution of microfluidic chip technology enables miniaturization, integration, and high-throughput protein analysis, overcoming the limitations of traditional protein analysis methodologies. These advancements in microfluidics not only enhance our understanding of cellular functions and disease mechanisms, but also lay the groundwork for developing reliable techniques for protein detection and analysis across a range of biotechnological applications. Protein preconcentration or enrichment using microfluidic chips has become a topic of significant interest in recent research. Various studies have explored different techniques to enhance preconcentration efficiency. A novel and efficient method for protein preconcentration using self-assembled gold nanoparticles in nano-interstices has been reported, offering a simple yet reliable approach that eliminates the need for high-voltage or complex fabrication processes [6]. The significance of this method lies in its ability to achieve protein enrichment through the exclusion-enrichment effect, demonstrating the potential to enhance sensitivity and accuracy in biochemical analyses. Yuan et al. developed a method utilizing field-amplified sample stacking (FASS) technology to achieve the concentration enrichment of charged samples [7]. Chen and Timperman introduced a protocol using microchannel pretreatment to facilitate hydrogel polymerization for enhanced sample enrichment and ionic current rectification in microfluidic devices [8]. Gholinejad et al. investigated the impact of electric fields and buffer concentration on ion concentration polarization for optimizing analyte preconcentration [9]. These studies collectively contribute valuable insights into improving protein preconcentration/enrichment techniques in microfluidic systems. Analyzing protein concentration is essential in various biological and biomedical applications. Advancements in microfluidic technology have prompted researchers to devise innovative ways to quantify protein concentrations on microfluidic chips. These chips, composed of a network of microchannels, enable manipulation of small volumes of biological samples such as proteins, DNA, and cells. They have revolutionized protein analysis by providing high-throughput, miniaturized, and cost-effective methods. The miniaturization of protein analysis on these chips has significantly improved sensitivity, speed, and accuracy, making them an appealing platform for protein quantification. Protein quantification on microfluidic chips involves several methods, each with its distinctive strengths and limitations. The Bradford assay is a prevalent technique that quantifies proteins by binding them to the Coomassie Brilliant Blue dye [10]. This method is quick, straightforward, and sensitive, although it can be influenced by interfering substances present in the sample. Spectrophotometry is another approach that measures protein quantities by assessing light absorbance at specific wavelengths, offering accuracy and sensitivity, but requiring meticulous calibration and potentially affected by interfering substances [11]. Fluorescence-based methods, such as fluorescence resonance energy transfer (FRET), offer high sensitivity and specificity, but demand careful optimization and can be influenced by fluorescent impurities [12]. Mass spectrometry (MS), a highly sensitive and specific technique, quantifies proteins by detecting ions based on their mass-to-charge ratio [13]. Despite its high accuracy, it requires sophisticated instrumentation and can be impacted by interfering ions. In the realm of microfluidic chips, a common method involves using fluorescent dyes to mark the target protein [6,14]. This is followed by capturing images using a fluorescence microscope. Subsequently, software is used to measure the fluorescence intensity, which aids in determining the protein concentration. However, this strategy requires a bulky and expensive fluorescence microscope to capture images, which is unsuitable for point-of-care testing. In light of these limitations, the present investigation aims to develop a compact detection system with a microspectrometer to monitor protein concentration in real-time by detecting the absorption spectrum of fluorescent labels. This approach seeks to overcome the constraints of traditional methods while maintaining the accuracy and sensitivity required for effective protein analysis.

## 2. Design and Working Principle

**Figure 1:**
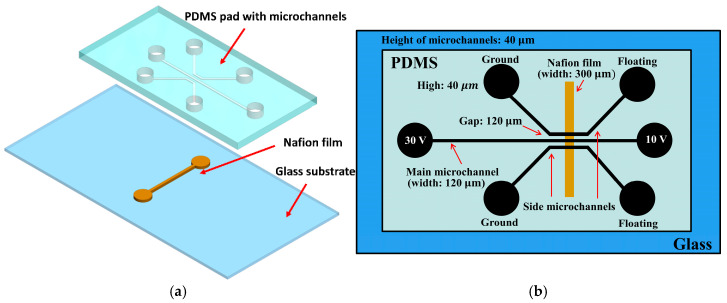
Design and structure of the proposed microfluidic chip for protein concentration. (**a**) Exploded view of the chip, showcasing the PDMS pad with microchannels and a straight Nafion film on the glass substrate. (**b**) Detailed illustration of the microfluidic channels’ design and dimensions, including one main microchannel and two side microchannels, with corresponding applied voltages.

**Figure 2:**
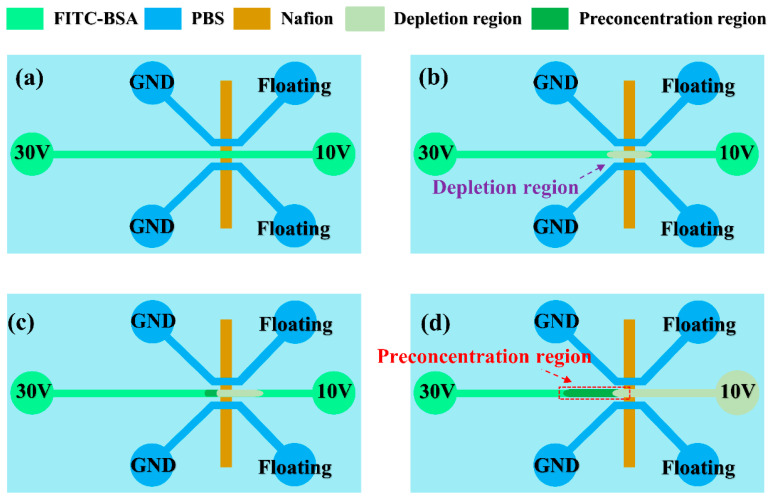
Schematic diagram of electrokinetic protein preconcentration operations in a microfluidic device. (**a**) Application of electric potential creates a localized electric field and concentration gradient. (**b**) Charged proteins, such as BSA, accumulate at the Nafion film interface due to selective permeability. (**c**) Bias voltages (30 V and 10 V) stimulate electroosmotic flow (EOF) to enhance protein accumulation. (**d**) Proteins stack in the preconcentration region at the intersection of the main microchannel and Nafion film.

The preconcentrator discussed in this work was constructed on a glass substrate, incorporating a Nafion film and PDMS. This device consists of two main layers: (1) a microfluidic channel layer made from PDMS and (2) a Nafion film layer positioned on a glass substrate. Figure 1 depicts the design and structure of this device. The device includes two side microchannels and a main microchannel for protein concentration, shown in Figure 1a. The microfluidic channels, each 120 μm wide and 40 μm high, were crafted in PDMS using standard soft lithography techniques [15]. The Nafion film was developed on the glass substrate utilizing the microflow patterning method [16]. In brief, a PDMS cover with a single microchannel (300 μm wide and 40 μm deep) was reversibly bonded to a glass substrate. Subsequently, 3 μL of 10 wt% Nafion liquid, diluted from a 20 wt% Nafion dispersion (D2020, Chemours, Wilmington, DE, USA) using alcohol and DI water, was injected into one open reservoir of the microchannel. The microchannel was filled using capillary forces. Most of the Nafion resin was removed from the microchannel by applying negative pressure through the syringe tubing at the other end of the microchannel. The glass substrate (soda-lime glass, Muto Pure Chemicals Co., Ltd., Tokyo, Japan, thickness: 1.0 mm) was heated on a hot plate at 95 °C for 10 min to increase adhesiveness with the Nafion film. After the PDMS cover was removed, only a thin layer of Nafion film remained on the glass substrate. Fluorescein isothiocyanate (FITC)-labeled bovine serum albumin (BSA) (Sigma-Aldrich, St. Louis, MO, USA) diluted in a 0.3 mM phosphate-buffered saline (PBS) solution (pH 7.4) at various concentrations was used to fill the microchannel via capillary force to demonstrate on-chip protein preconcentration. Figure 2 presents a schematic diagram of electrokinetic protein preconcentration operations. Protein preconcentration in this microfluidic device occurs through ion concentration polarization, utilizing a Nafion film integrated into the chip. When an electric potential is applied across the microchannels, ions migrate toward the electrodes, creating a localized electric field and concentration gradient. This gradient causes charged proteins, such as BSA, to accumulate at the interface between the Nafion film and the main channel. The Nafion film’s selective permeability allows for specific ions to pass while retaining proteins, thereby creating a concentrated protein zone [17,18]. The depletion regions on both sides of the Nafion film extend when DC voltages of 30 V and 10 V are applied to the two anodic side reservoirs. This occurs while the reservoirs at the side microchannels are grounded and the rest are floating, as shown in Figure 2a,b. Bias voltages of 30 V and 10 V are applied to both sides of the main microchannel to stimulate electroosmotic flow (EOF) and accumulate proteins, as shown in Figure 2c. The proteins then accumulate and stack in the preconcentration region near the intersection of the main microchannel and the Nafion film, as illustrated in Figure 2d. Voltage distribution plays a vital role in protein preconcentration efficiency. The applied voltage generates an electric field that drives charged biomolecules and electrolyte ions, causing concentration polarization at the Nafion film. This process creates an ion imbalance, depleting ions on the anodic side while enriching them on the cathodic side. Near the film, the accumulated positive ions form a nonequilibrium electrical double layer (EDL), enhancing EOF in the anodic compartment. This EOF combines with the electrophoretic flow of negatively charged proteins to concentrate them at the anodic side. Furthermore, variations in voltage distribution create localized electric fields that generate vortex-like flows, enhancing biomolecule transport. Therefore, optimizing voltage distribution is critical for achieving maximum preconcentration efficiency [17]. The voltages of 30 V and 10 V were chosen based on preliminary results showing their effectiveness in system performance. These higher voltages generate stronger EOF, crucial for driving proteins toward the Nafion film and enhancing concentration. These voltage levels significantly improve enrichment efficiency, especially for low-concentration samples. While lower voltages are theoretically possible, our initial experiments showed that they did not generate sufficient EOF to achieve desired concentration levels. Lower voltages led to slower accumulation rates, which could compromise detection sensitivity and accuracy. Future studies could explore how different voltage levels affect system performance to optimize the electrokinetic preconcentration process and improve protein detection sensitivity. The electrokinetic protein preconcentration method is significantly influenced by the isoelectric point of the proteins and the pH value of the buffer. Our previous research [19] has shown that proteins exhibit enhanced concentration efficiency at pH values above their isoelectric point, as this condition increases their negative charge, facilitating more effective electrokinetic concentrating. Conversely, at pH values below the isoelectric point, proteins may become less negatively charged, which can hinder their concentration. Furthermore, it is important to note that extreme pH values can lead to protein denaturation, thereby limiting the applicability of this enrichment technique. The selectivity of the Nafion membrane is influenced by various factors, including operational duration, protein concentration, and applied voltage. In the current study, the stability of the Nafion membrane was observed under optimal operating conditions throughout the experimental timeframe. While prolonged usage may potentially lead to reduced selectivity due to protein fouling or alterations in the ionic environment, it may be beneficial for future studies to monitor the selectivity of the Nafion membrane over extended periods. This approach will provide deeper insights into its long-term performance and stability in protein enrichment applications.

## 3. Experimental Setup

**Figure 3:**
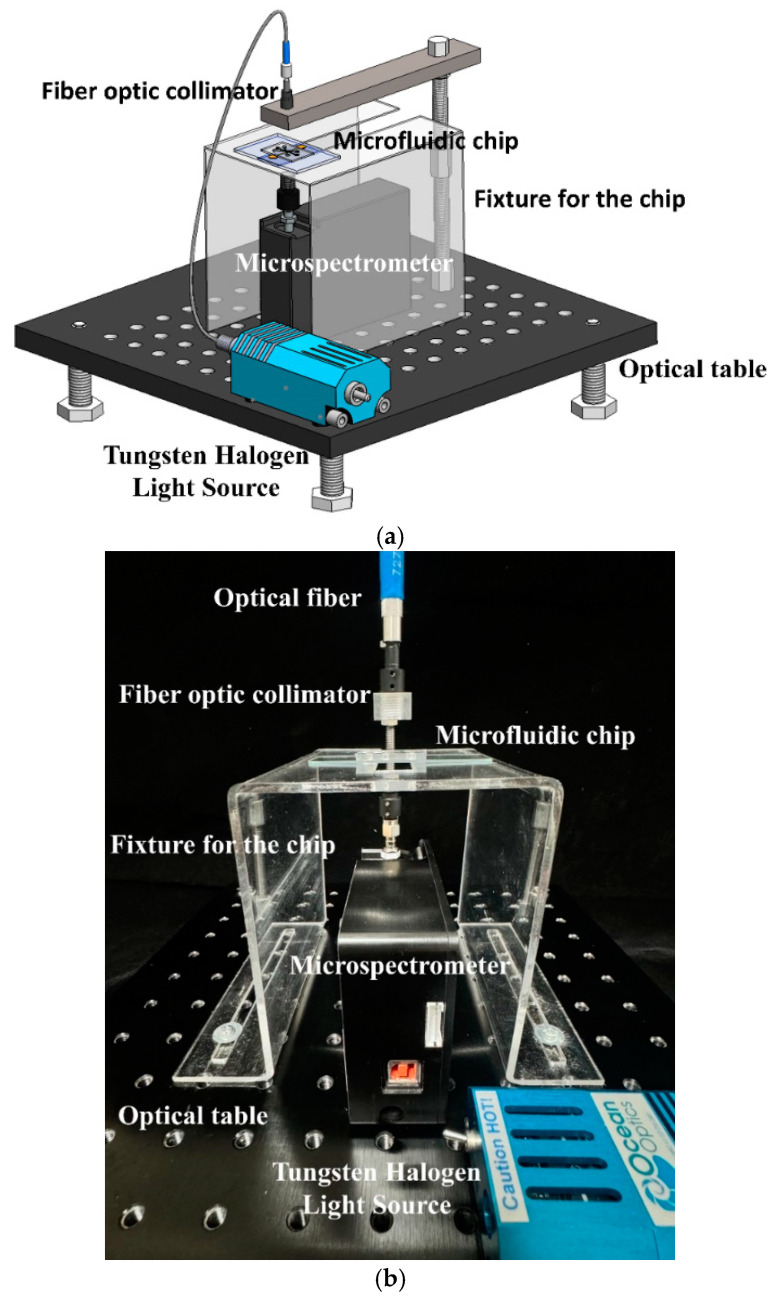
(**a**) Schematic diagram of the microspectrometer system for detecting protein preconcentration and (**b**) a photograph of the assembled experimental setup.

In our previous report [20], protein preconcentration was observed and recorded using an inverted fluorescence microscope equipped with a CCD camera connected to a computer running imaging software. Quantification of the fluorescent intensities emitted by enriched FITC-labeled BSA was performed using image analysis software capable of assessing pixel density. For the present study, a microspectrometer was adopted for protein concentration measurement. A system utilizing the microspectrometer to detect protein concentration was designed and implemented. This approach represents a shift towards more compact and real-time detection methods, potentially overcoming limitations associated with traditional fluorescence microscopy techniques in protein analysis. Figure 3a illustrates the design of this system, which consists of a microspectrometer (Model SE2030-025-FUV, OTO Photonic Inc., Hsinchu City, Taiwan), a tungsten halogen light source (Model # LS-1, Ocean Optics Inc., Dunedin, FL, USA), and a fiber optic collimator placed on an optical table. Figure 3b shows a photograph of the assembled experimental setup. All components have been properly installed and connected according to the schematic design shown in Figure 3a. However, the light source from the optic collimator, with an aperture of around 5 mm, illuminates a microchannel that is only 120 µm wide. This significant size difference means that most of the light energy falls outside the channel, reducing the effectiveness of the irradiation on the sample and affecting measurement accuracy. To address this issue, this study designed a slot collimator to prevent the light source from illuminating outside the channel, thus enhancing the measurement accuracy of the microspectrometer.

**Figure 4:**
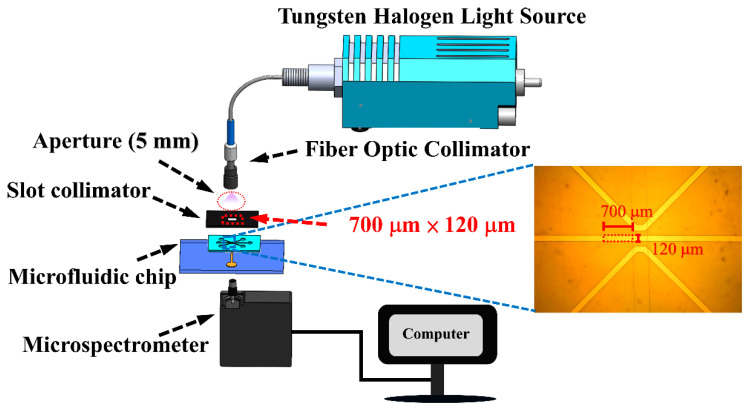
Design of the entire experimental setup including the slot collimator and microchannel dimensions.

Figure 4 illustrates the overall design of the experimental setup, emphasizing the slot collimator and the dimensions of the microchannel. The slot collimator, measuring 120 μm in width and 700 μm in length, is directly affixed to the microfluidic chip to optimize light delivery into the narrow microchannel. This configuration minimizes light loss and enhances the effective irradiation of the sample, which consists of proteins labeled with fluorescent dyes confined to the 120 μm wide microchannel. Given that the light source from the optical collimator has an aperture of approximately 5 mm, it illuminates a microchannel that is significantly narrower. Consequently, only a small fraction of the incident light interacts with the sample, leading to considerable measurement errors and a relatively low absorbance. To address this issue, the design of the slot collimator ensures that light is directed exclusively into the microchannel. This targeted illumination increases the effective interaction area between the light and the proteins labeled with fluorescent dyes within the microchannel, thereby enhancing measurement sensitivity and providing more accurate absorbance readings. This approach aims to significantly improve measurement precision in the microspectrometer by aligning the light source more effectively with the dimensions of the microchannel. The primary absorption peak of most proteins, including BSA, occurs at 280 nm, attributed to the presence of tryptophan and tyrosine residues [21]. This wavelength is optimal for measuring BSA concentrations using UV light sources. However, in this study, soda-lime glass was employed as the substrate material, which has significant UV absorption at 280 nm [22,23,24]. This characteristic leads to substantial attenuation of light intensity at this wavelength as it passes through the glass substrate, complicating effective transmission. While quartz glass, composed mainly of silicon dioxide (SiO_2_), is ideal for UV measurements at 280 nm due to its low absorption in this range, its high cost limits its practicality for widespread applications. Consequently, this study aims to establish a cost-effective microspectrometer-based measurement platform utilizing fluorescein isothiocyanate (FITC)-labeled BSA for system validation and testing. This approach offers an alternative method for protein detection and quantification, balancing performance with cost considerations. FITC, a small organic fluorescent dye with a molecular weight of 389.4 g/mol, exhibits optimal light absorption within a spectral range from 490 to 495 nm. Upon excitation, FITC emits fluorescence in a range from 520 to 530 nm, producing a vivid yellow-green color that is highly perceptible to the human eye. This distinctive fluorescence characteristic makes FITC a widely utilized probe in biomedical research applications [25,26]. The absorption characteristics of the FITC-BSA conjugate are influenced by several factors, including the labeling ratio, concentration of FITC, and molecular interactions, which can lead to shifts and broadening in the absorption spectrum [27]. It was found in our experiments that the FITC-BSA system used in this study, dissolved in 0.3 mM PBS solution, exhibited a peak absorption wavelength of 491 nm. Consequently, this wavelength was selected for subsequent measurements in the current investigation.

## 4. Results and Discussion

**Figure 5:**
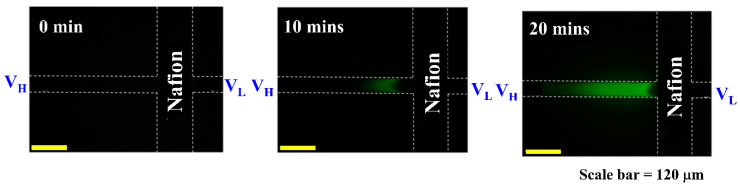
Timelapse fluorescence microscopy of FITC-labeled BSA preconcentration. Images show the spatial distribution of 2 μM FITC-BSA in 0.3 mM PBS after initiation of preconcentration. Images were acquired using an inverted fluorescence microscope. Scale bar: 120 µm.

**Figure 6:**
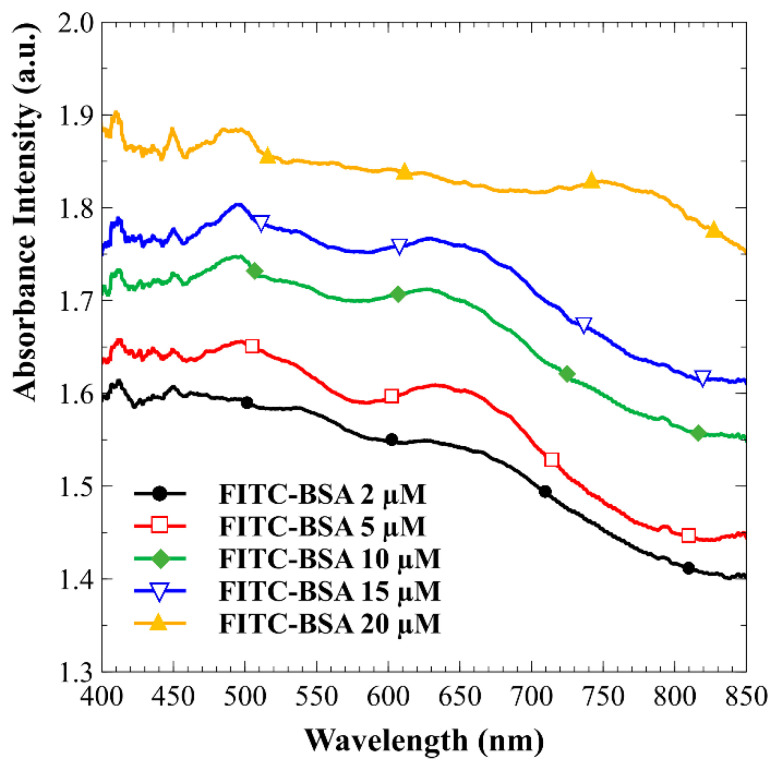
Absorption spectra of FITC-BSA at varying concentrations.

**Figure 7:**
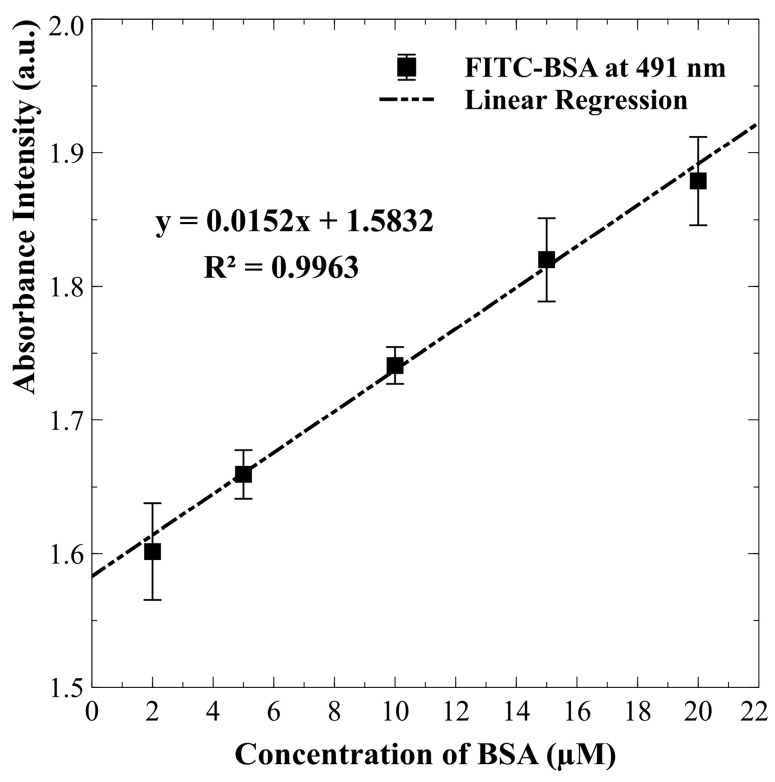
Calibration curve illustrating the relationship between protein concentration and the absorption spectral values of FITC at a wavelength of 491 nm. The concentration range tested ranged from 2 to 20 μM. Error bars represent the standard deviation of measurements obtained from at least three independent replicates for each condition.

A series of experiments was initiated to investigate the effects of varying initial protein concentrations on the efficiency of protein concentration over time. The proposed detection system, equipped with a microspectrometer, was utilized to systematically monitor the absorption spectra of fluorescent labels at different time intervals. Figure 5 illustrates the progression of FITC-labeled BSA accumulation over time. The images reveal a gradual increase in the concentration and spatial distribution of proteins within the 0.3 mM PBS solution. Over 20 min, a marked enhancement in FITC-BSA presence becomes evident, confirming the effectiveness of the preconcentration process. This timelapse sequence illustrates the dynamics of FITC-BSA concentration in the experimental setup. Absorption measurements were conducted to investigate the optical absorption properties of FITC-BSA at varying concentrations. The proposed microspectrometer system effectively measured light absorption across a range of wavelengths for each concentration of FITC-BSA solution. As illustrated in Figure 6, the resulting absorption spectra demonstrate a clear correlation between concentration and absorption intensity. Calibration curves for protein concentration and the absorption spectral values of FITC at a wavelength of 491 nm were established using the measurement system developed in this study. The experimental results, illustrated in Figure 7, reveal an R^2^ value of 0.9963 from the linear regression analysis. This high R^2^ value indicates a strong linear relationship between protein concentration and the corresponding absorption spectra. Such a result suggests that the detection system is capable of accurately quantifying protein concentrations within the tested range, thereby confirming its reliability for real-time monitoring applications.

**Figure 8:**
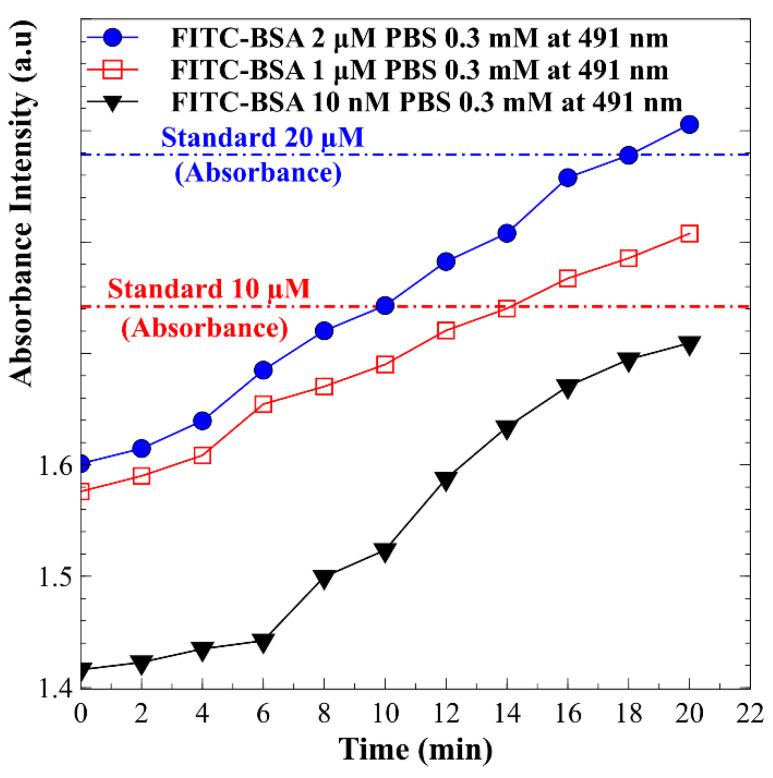
Variations in absorption peak values of BSA protein samples with initial concentrations of 2 µM, 1 µM, and 10 nM over a 20 min period.

Subsequent experiments were conducted utilizing BSA protein samples at initial concentrations in the micromolar range, specifically 2 μM and 1 μM. The results, illustrated in Figure 8, depict the changes in absorption peak values over a 20 min period. It was observed that the concentration of BSA increased with time. At the conclusion of the 20 min interval, the concentrations of BSA corresponding to the initial values of 2 μM and 1 μM were determined to have increased by factors of 10.52 and 14.74, respectively, as inferred from the calibration curve presented in Figure 7. Further investigations focused on lower concentration samples, employing BSA with an initial concentration of 10 nM. The results also shown in Figure 7 reveal a distinct trend of increasing protein concentration over time. By the end of the 20 min period, the BSA concentration had increased significantly, reaching an enhancement factor of 833 times its initial value. This substantial increase underscores the sensitivity and efficacy of the current detection system in monitoring protein concentrations, even at low initial levels. These findings highlight the potential applications of this methodology in contexts requiring precise quantification of low-concentration proteins. Using the same preconcentration chip for protein enrichment, it was observed that lower initial protein concentrations yielded higher concentration factors. This trend was particularly pronounced when comparing nanomolar and micromolar levels. At nanomolar concentrations, concentration factors approaching one thousand were achieved, whereas at micromolar concentrations, the factors typically remained in the low tens. Several factors contribute to this phenomenon. At lower initial concentrations, the detection capabilities of the system become more sensitive, allowing for a more pronounced relative increase in concentration as proteins are processed. Additionally, the dynamics of protein interactions and binding affinities tend to favor accumulation at lower concentrations, resulting in enhanced concentration ratios. The overall efficiency of the concentration process is often greater at lower initial levels, as the overlapping electric double layers within the nanopores promote protein accumulation while minimizing competition among molecules for binding sites. This mechanism leads to more effective concentration outcomes. This relationship underscores the ability of the proposed detection system to accurately measure low initial concentration samples, effectively elevating their concentrations as desired. The motivation behind the design of the preconcentration chip is to enhance detection performance by increasing extremely low sample concentrations within a specific area, thereby facilitating more accurate and sensitive measurements.

## 5. Conclusions

The development of the microspectrometer-enabled microfluidic protein enrichment chip represents a significant advancement in protein analysis, providing a novel method for real-time monitoring of protein concentrations that effectively addresses key challenges in the field. This innovative system integrates a microspectrometer with a microfluidic platform, enhancing sensitivity and specificity in protein detection while eliminating the need for bulky and expensive traditional fluorescence microscopy. The design features a Nafion film and PDMS microchannels which facilitate efficient protein enrichment through electrokinetic methods, thereby improving the overall efficiency of the preconcentration process and minimizing sample loss. Experimental results confirm the ability of the proposed system to accurately measure low protein concentrations, establishing a strong linear relationship between protein concentration and the corresponding absorption spectra, which is crucial for detecting low-abundance proteins relevant to disease diagnosis. The capability of monitoring protein concentrations in real-time provides valuable insights into the kinetics of protein interactions and the efficiency of the enrichment process, allowing for dynamic analyses that can inform the optimization of experimental conditions. While the immediate findings highlight the effectiveness of this technology in protein analysis, the potential applications in clinical diagnostics and research are noteworthy, as this system could facilitate the rapid detection of disease-specific biomarkers. It is worth noting that FITC-labeled BSA was diluted in a 0.3 mM PBS solution (pH 7.4) to establish a controlled environment for evaluating protein concentration detection effectiveness with a microspectrometer herein. Using this simplified matrix enables clearer assessments of detection performance, as complex biological samples typically present challenges due to variability and interfering substances. Research has shown that biological matrix complexity can impair protein separation efficiency, requiring effective sample preparation and fractionation techniques to enhance analytical results [28]. Methods such as ultrafiltration and gel electrophoresis enhance protein recovery and resolution from complex mixtures, making them applicable for real-world biological samples including saliva, blood, and urine [29]. Although the current findings derive from a simplified model system, implementing appropriate sample preparation strategies should enable the successful application of this method to more complex biological matrices. Overall, this research lays the groundwork for future developments in protein analysis technologies, with the promise of improving diagnostic capabilities and enhancing our understanding of protein dynamics in various biological contexts.

## Data Availability

The data presented in this study are available on request from the corresponding author.
